# A Further Look at Porcine Chromosome 7 Reveals *VRTN* Variants Associated with Vertebral Number in Chinese and Western Pigs

**DOI:** 10.1371/journal.pone.0062534

**Published:** 2013-04-24

**Authors:** Yin Fan, Yuyun Xing, Zhiyan Zhang, Huashui Ai, Zixuan Ouyang, Jing Ouyang, Ming Yang, Pinghua Li, Yijie Chen, Jun Gao, Lin Li, Lusheng Huang, Jun Ren

**Affiliations:** Key Laboratory for Animal Biotechnology of Jiangxi Province and the Ministry of Agriculture of China, Jiangxi Agricultural University, Nanchang, People’s Republic of China; The Roslin Institute, University of Edinburgh, United Kingdom

## Abstract

The number of vertebrae is an economically important trait that affects carcass length and meat production in pigs. A major quantitative trait locus (QTL) for thoracic vertebral number has been repeatedly identified on pig chromosome (SSC) 7. To dissect the genetic basis of the major locus, we herein genotyped a large sample of animals from 3 experimental populations of Chinese and Western origins using 60K DNA chips. Genome-wide association studies consistently identified the locus across the 3 populations and mapped the locus to a 947-Kb region on SSC7. An identical-by-descent sharing assay refined the locus to a 100-Kb segment that harbors only two genes including *VRTN* and *SYNDIG1L*. Of them, *VRNT* has been proposed as a strong candidate of the major locus in Western modern breeds. Further, we resequenced the *VRTN* gene using DNA samples of 35 parental animals with known QTL genotypes by progeny testing. Concordance tests revealed 4 candidate causal variants as their genotypes showed the perfect segregation with QTL genotypes of the tested animals. An integrative analysis of evolutional constraints and functional elements supported two *VRTN* variants in a complete linkage disequilibrium phase as the most likely causal mutations. The promising variants significantly affect the number of thoracic vertebrae (one vertebra) in large scale outbred animals, and are segregating at rather high frequencies in Western pigs and at relatively low frequencies in a number of Chinese breeds. Altogether, we show that *VRTN* variants are significantly associated with the number of thoracic vertebrae in both Chinese and Western pigs. The finding advances our understanding of the genetic architecture of the vertebral number in pigs. Furthermore, our finding is of economical importance as it provides a robust breeding tool for the improvement of vertebral number and meat production in both Chinese indigenous pigs and Western present-day commercial pigs.

## Introduction

The number of vertebrate is a phenotypic trait with high heritability estimates of 0.60 to 0.62 in domestic pigs [1], [2]. The porcine vertebrae consist of morphologically differentiated formulae: cervical, thoracic, lumbar, sacral and caudal vertebrae. The numbers of cervical and sacral vertebrae are always fixed at 7 and 4 except for rare cases in pigs [3], [4] as in other mammals, showing an evidence of evolutionary constraint [5]. In contrast, the number of thoracic and lumbar vertebrae varies considerably in pigs. Wild boars, the ancestors of domestic pigs, have uniformly 19 thoracic and lumbar vertebrae. Most Chinese indigenous breeds show 19 or 20 thoracolumbar vertebrae [6]. Comparatively, Western modern breeds, such as Duroc, Landrace and Large White, have more (n = 21–23) thoracic-lumbar vertebral formulae [1]. The increased vertebral number is likely caused by the long-term intense selection on enlarged size in these breeds.

The number of vertebrae is an economically important trait in pigs as it is associated with body size and carcass length. It has been estimated that one extra vertebra can expand the carcass length of about 80 mm [3]. Therefore, deciphering the genetic basis of swine vertebral number variation will not only improve our understanding of vertebral developmental biology in mammals but also benefit the selective breeding for larger body size in the pig industry.

Several studies have reported quantitative trait loci (QTL) for vertebral number in pigs by genome scans on the basis of microsatellite markers. Two genome-wide significant QTL were first detected on pig chromosomes (SSC) 1 and 2 in a Meishan × Göttingen cross [7]. Subsequently, the QTL on SSC1 has been repeatedly evidenced in different studies, explaining the variation in one vertebra [8]–[10]. Mikawa et al [11] further show that *NR6A1*c.134 G>A is a promising candidate causal variant underlying the major QTL. Besides, another genome-wide significant QTL for the number of thoracic vertebrae has been consistently identified on SSC7 by different investigators [9], [10], [12], [13]. More recently, the *VRTN* gene was proposed to be a strong candidate of the SSC7 QTL in Western commercial breeds [14]. However, the causality of *VRTN* needs more supporting evidence. It is worth noting that the QTL has also been detected in Chinese breeds [10]. Therefore, additional efforts are required to address if *VRTN* is also the responsible gene for the QTL detected in Chinese breeds. Moreover, the causative variant of *VRTN* has not yet been defined and thus needs further investigation.

In our previous report [10], we detected the major QTL for the number of thoracic vertebrae on SSC7 using a genome scan with 194 microsatellite markers in a White Duroc × Erhualian F_2_ resource population. We found that the QTL affects the thoracic vertebral number in both Western and Chinese pigs. The aim of this study was to characterize the responsible gene and the most likely causal variant for the SSC7 QTL by a series of genetic analyses including genome-wide association studies (GWAS), identical-by-descent (IBD) sharing mapping, genetic concordance test, evolutional constraint assay and association analysis in a large scale outbred samples.

## Materials and Methods

### Ethics Statement

All the procedures involving animals are in compliance with the care and use guidelines of experimental animals established by the Ministry of Agriculture of China. The ethics committee of Jiangxi Agricultural University specifically approved this study.

### Animals and Phenotype Recording

In this study, experimental animals were from four pig populations including the White Duroc × Erhualian F_2_ intercross, an Erhualian × Tongcheng F_2_ resource population, a Chinese Sutai purebred population and a Western three-way hybrid (Duroc × Landrance × Large White, DLL) commercial population. The White Duroc × Erhualian cross was developed and managed as described by Guo et al. (2009) [15]. In brief, two White Duroc sires and 17 Erhualian dams were mated to produce F_1_ animals. A total of 1912 F_2_ animals were generated in six batches by intercrossing 9 F_1_ boars with 59 F_1_ sows avoiding full-sib mating. Of the 1912 animals, 918 individuals were slaughtered for phenotype recording at the age of 240±3 days. In the Erhualian × Tongcheng F_2_ pedigree, one Chinese Erhualian boar was mated to one Chinese Tongcheng sow, and 2 F_1_ boars and 7 F_1_ sows were then intercrossed to produce 61 F_2_ individuals that were slaughtered at 60 days of age. Sutai is a Chinese synthetic breed that was originally produced by crossing Chinese Taihu and Western Duroc (50% Taihu and 50% Duroc) [16]. In the current study, 4 Sutai boars were mated with 55 Sutai sows to produce 461 offspring, of which 435 were slaughtered at 240±3 days of age. Animals of the three experimental populations were all raised in the research farm at Jiangxi Agricultural University in Nanchang, China. In addition, 1403 pig samples were collected from 9 three-way hybrid (DLL) populations in a commercial slaughterhouse in Nanchang. After slaughter, all animals were recorded for the number of thoracic and lumbar vertebrae.

### SNP Chip and Genotyping

Genomic DNA was extracted from ear tissue of each animal using a standard phenol/chloroform method. DNA quality was determined by a Nanodrop-100 spectrophotometer (Thermo Fisher, USA). All eligible DNA samples were diluted to a final concentration of 50 ng/µl. The samples were genotyped for 62163 SNPs on the Porcine SNP 60K Beadchips (Illumina, USA) according to the supplier’s protocol. The same quality control criteria were applied for the SNP data of each population by the check.marker function of GenABEL [17]. Animals with SNP call rates ≥95% and familial Mendelian error rates ≤0.1, and SNPs with call rates ≥95%, minor allele frequencies (MAF) ≥0.1 and significance levels of deviation from Hardy-Weinberg equilibrium ≤10^−6^ were included for further statistical analysis.

### GWAS Mapping

A mixed model-based single-locus regression analysis was performed for GWAS mapping using GenABEL, an R library for whole genome association analysis [17]. The analysis adjusted population stratification by modeling similarities between individuals on the basis of genome-wide SNP data. For the meta-analysis of GWAS, the statistical χ^2^ values at each locus in each experimental population were summed to calculate new χ^2^ values with a freedom degree of 3. Bonferroni corrected *P*-values were adopted for the genome-wide significance threshold that was set as 0.05/N, where N is the number of informative SNPs in the data set.

### Haplotype Sharing Analysis

The QTL genotypes of F_1_ sires in two F_2_ populations and progenitor boars in Sutai pigs were first determined by the marker-assisted segregation analysis as described in Nezer et al [18]. Briefly, the likelihood ratio of sire’s QTL genotypes for homozygotes (*QQ* or *qq*) or heterozygotes (*Qq*) was calculated using offspring’s phenotypic data by grouping the “Left” chromosome or “Right” chromosome separately. A Z-score log10 statistics was then obtained by comparing the likelihood of heterozygotes to the likelihood of homozygotes. Sires were considered to be *Qq* when Z>2, *QQ* or *qq* when Z<−2, and unknown QTL genotypes when Z in the interval of −2 to 2. Further, the QTL genotypes of F_0_ sires in the F_2_ populations were judged by the Mendelian inheritance and multiple comparison tests against the deduced *Q*-chromosomes of F_1_ sires using a classical *t*-test.

Haplotypes of sires with successfully deduced QTL genotypes were reconstructed with SimWalk2 v2.91 [19] under default setting based on the pedigree information. All *Q*-bearing chromosomes were examined for their shared haplotypes to narrow down the QTL position. To improve haplotype-sharing resolution, high density markers in addition to the 60K SNP data were characterized in the targeted region by comparative sequencing of 35 parental samples with known QTL genotypes. Genomic DNA was amplified with primers listed in **Table S1** at optimal annealing temperatures. PCR products were directly sequenced with PCR primers on a 3130XL Genetic Analyzer (Applied Biosystem, USA). Variants were then recorded by the alignment of sequence reads.

### Resequencing of the *VRTN* Gene

To resequence the porcine *VRTN* gene, a set of primers (**Table S1**) were used to amplify genomic DNA of the 35 parental pigs. Amplification was performed in a routine way with 1.5 mM of MgCl_2_ and optimal annealing temperatures. The resulting PCR products covered a ∼30-kb region harboring the *VRTN* gene. All amplicons were bi-directionally sequenced with original PCR primers on the 3130XL Genetic Analyzer (Applied Biosystem, USA). Variants were excluded as the causal mutation if their genotypes were not concordant with the QTL genotypes in the sire samples.

### Analyses of Evolutional Constraints and Functional Significance of *VRTN* Candidate Causal Variants

Genomic sequences of human, mouse, cattle, dog and horse *VRTN* genes were retrieved from the NCBI nucleotide database (http://www.ncbi.nlm.nih.gov/gene/?term=VRTN). The MultiPipMaker software [20] was implemented to analyze evolutionary constraints of *VRTN* candidate causal variants. Further, Genomic Evolutionary Rate Profiling (GREP) alignment [21] based on 30 eutherian mammals were determined for the most likely causal variants via the Ensembl Genome Browser (www.ensembl.org/). Functional elements were predicted for the regions harboring candidate causal variants by an integrative genomic analysis using the UCSC Genome Browser at http://genome.ucsc.edu/.

### Association and Genetic Variability of the Most Likely Causal Variants in Outbred Populations

Two most likely causal variants, g.19034A>C and g.20311_20312ins291 (GenBank accession no. AB554652.1), were genotyped on 435 Sutai pigs and 192 Western purebred pigs. The genotypes of g.19034A>C were detected by a TaqMan SNP assay (Primers F5/R5 and the corresponding probe, **Table S1**) on a 7900HT Fast Real-Time PCR System (Applied Biosystem, USA), For g.20311_20312ins291, the genotypes were judged using a PCR-based test (Primers F6/R6, **Table S1**). PCR products were separated by 2% agrose gel electrophoresis and the genotypes were visually recorded according to the length of amplicon. The *ins* allele was represented by amplicons of 411 bp and the wild-type allele by amplicons of 120 bp (**Figure S1**). Moreover, 1403 Western three-way hybrid pigs and 1371 purebred pigs from 19 diverse breeds and wild boars were genotyped for g.20311_20312ins291. Association of the variants with the number of thoracic vertebrae was performed on the Sutai and Western hybrid animals by a classical *t*-test. Frequencies of the *ins* (*Q*) allele increasing vertebral number were calculated for each purebred population.

## Results

### GWAS Mapped the Major QTL to a 947-Kb Region on SSC7

**Figure 1:**
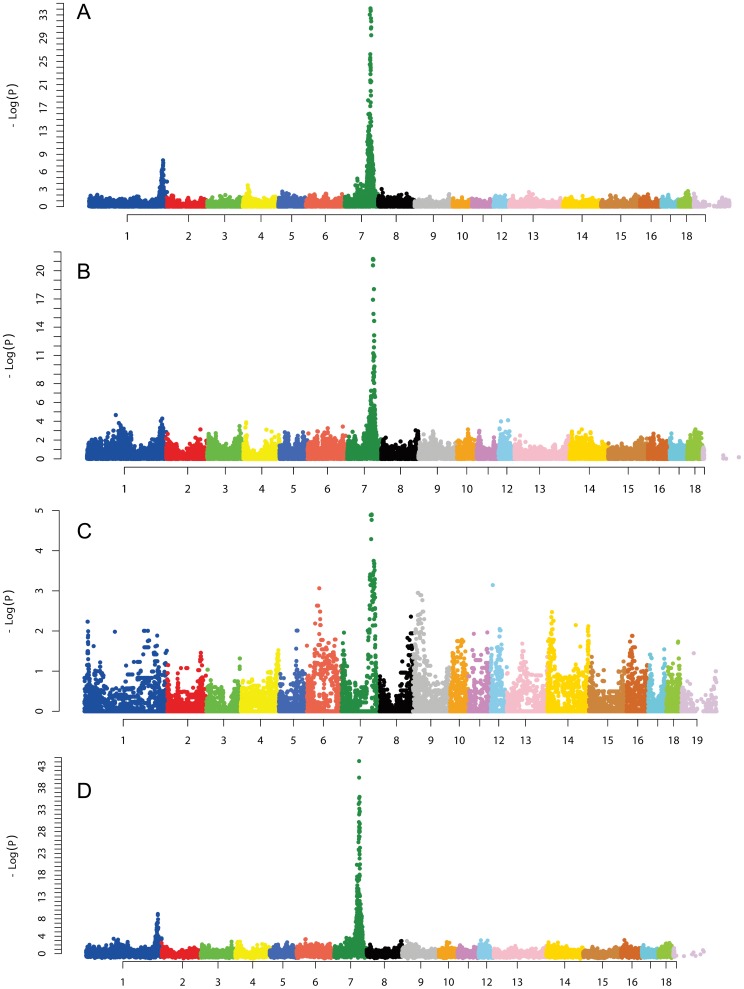
GWAS map the major QTL for the number of thoracic vertebrae to SSC7. GWAS were performed on the White Duroc × Erhualian F_2_ intercross (A), Sutai pigs (B), Erhualian × Tongcheng F_2_ intercross (C) and meta-analysis (D). Negative log10 *P*-values of all SNPs are plotted against position on each pig chromosome in the y-axis. Chromosomes are shown in different colors for clarity in the x-axis. Log (1/*P*) values of more than 5 are genome-wide significant.

In the present study, we conducted GWAS for vertebral number in 3 distinct populations. A total of 39448, 43760 and 20509 informative SNPs were filtered for GWAS on the White Duroc × Erhualian F_2_ intercross, Sutai pigs and the Erhualian × Tongcheng F_2_ intercross, respectively. All experimental animals were successfully genotyped for the 60K chips. Consistently, we detected the most significant SNPs on SSC7 across the three populations (**Figure 1**). To determine whether the locus affects numeric variation in thoracic vertebrae or lumbar vertebrae, we performed GWAS for the two phenotypic traits separately in these populations. We found that the locus specifically affects the number of thoracic vertebrae in contrast to its negligible effect on lumbar vertebral formula (**Figure S2**), which is consistent with our previous QTL mapping result [10]. The top SNPs had a strong additive effect of approximate 0.5 thoracic vertebrae per allele (**Table S2**). In the White Duroc × Erhualian intercross (**Figure 1A**), the strongest SNP was H3GA0022664 at 103.91 Mb on SSC7 (*Sscrofa*10.2), which is located in intron 4 of the *PROX2* gene. In the Sutai population (**Figure 1B**), GWAS identified the most significant SNP at 102.46 Mb on SSC7. The SNP MARC0113727 resides in the *NUMB* gene. The most prominent SNP in the Erhualian × Tongcheng F_2_ population was CASI0006750 at 115.51 Mb that is located in an interval region flanked by the *FLRT2* and *GALC* genes (**Figure 1C**). The consistent GWAS results strongly indicate that a common variant underlies the QTL detected in these populations. We also performed a meta-analysis of GWAS for vertebral number across the three populations. The most significant finding was H3GA0022664 at 103.91 Mb, the top SNP evidenced in the White Duroc × Erhualian intercross. The SNP showed much higher significance level (LOD = 41) in the meta-analysis with combined data (**Figure 1D**). Two second significant SNPs were ASGA0035500 at 103.57 Mb and MARC0030523 at 101.86 Mb with LOD values of 38 and 37, respectively. Further, we defined the 95% confidence intervals (CI) of the major locus by LOD dropoff 2 from the strongest SNP. According to this criterion, the critical regions were 101.86–105.75, 102.46–104.31 and 103.37–117.04 Mb on SSC7 in the White Duroc × Erhualian intercross, Sutai pigs and Erhualian × Tongcheng intercross populations, respectively. The ∼947-Kb (103.37–104.31 Mb) region of overlap between the 95% CIs is of great interest as it defines the most likely QTL position harboring the responsible gene. For subsequent analysis, we focused on the ∼947-Kb segment.

### IBD Analysis Pinpointed *VRTN* as the Responsible Gene

**Figure 2:**
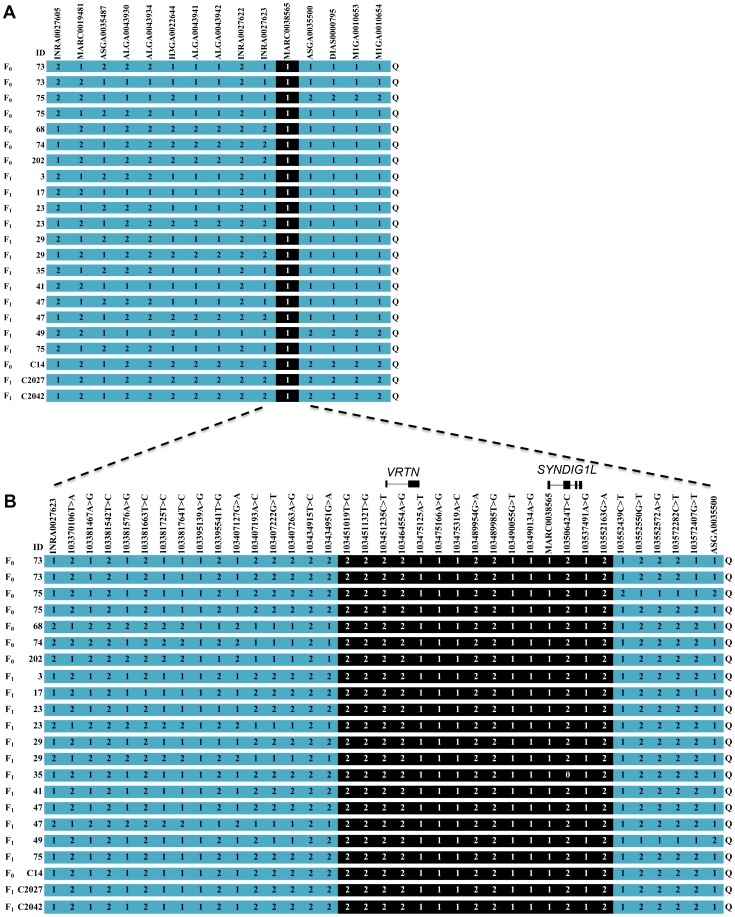
All Q-bearing chromosomes for increased vertebral number share a 100-Kb region harboring the *VRTN* gene. The IBD critical regions defined by the 60 K SNP data (panel A) and the characterized SNPs (panel B) are indicated by shaded boxes. Two annotated genes, *VRTN* and *SYNDIG1L*, are located in the IBD region. SNP alleles are shown by 1 and 2 for the major and minor alleles, respectively. The characterized SNPs shown in panel B are numbered according to their locations in the current pig genome assembly (*Sscrofa*10.2). Identities of animals carrying the *Q*-chromosome are given in the left axis. Animals C14, C2027 and C2042 are from the Erhualian × Tongcheng F_2_ intercross, and the others are from the White Duroc × Erhualian F_2_ intercross.

To perform IBD sharing analysis for fine mapping of the SSC7 QTL, we first determined the QTL genotypes of 12 F_1_ sires in the two F_2_ populations and 4 Sutai progenitor boars by marker-assisted segregation (**Table 1**). Further, we judged the QTL status of 19 founder sires and dams in the F_2_ populations by multiple comparisons of targeted chromosomes with the reference *Q* (number-increase) or *q* (wild-type) chromosomes of F_1_ sires. In total, we identified 22 *Q* alleles and 44 *q* alleles (**Table 1** **,** **Figure 2**). Haplotype reconstruction allowed us to identify the chromosomal region shared by all *Q* chromosomes. In the 947-Kb critical region, all 22 *Q*-bearing chromosomes across the three populations shared only one region of 128-Kb defined by two SNPs: INRA0027623 and ASGA0035500 (**Figure 2A**). To exclude the possibility of false positive IBD, we developed high density markers around the putative IBD region by comparatively sequencing 13 amplicons of the 35 individuals with known QTL genotypes. A final set of 35 informative markers was identified around the region. Haplotype reconstruction analysis clearly showed that all *Q*-bearing chromosomes share a unique haplotype of 100-Kb corresponding to 15 variants. The IBD region harbors only two annotated genes: *VRTN* and *SYNDIG1L* (**Figure 2B**). Of them, *SYNDIG1L* seem to be implicated in Huntington diseases in rodent models [22], while *VRTN* has been proposed as a strong candidate of the QTL for vertebral number [14]. Hence, our finding supports the assumption that *VRTN* is the responsible gene for the SSC7 QTL.

**Table 1:** Determination of QTL genotypes of founder animals by the *t*-test or marker assisted segregation analysis in three experimental populations.

			Average no. of thoracic vertebrae^b^ (no. of individuals)				
Population^a^	Generation	Animal	Left chromosome	Right chromosome	t-test	*Z*-test^c^	QTL genotype
WE	F_1_	3	15.33±0.50 (79)	14.50±0.60 (78)		13.46	Qq
		17	15.37±0.62 (57)	14.61±0.49 (49)		8.07	Qq
		23	15.30±0.48 (10)	15.18±0.60 (11)		−4.04	QQ
		29	15.34±0.60 (47)	15.27±0.49 (52)		−2.08	QQ
		35	15.33±0.66 (39)	14.49±0.51 (39)		6.34	Qq
		41	15.28±0.58 (87)	14.64±0.53 (83)		10.42	Qq
		47	15.35±0.63 (26)	15.40±0.50 (20)		−2.08	QQ
		49	15.22±0.64 (45)	14.51±0.56 (37)		4.73	Qq
		75	15.39±0.62 (56)	14.67±0.52 (45)		6.95	Qq
	F_0_	038	14.50±0.40 (30)	14.40±0.27 (10)	<0.0001		qq
		052	14.63±0.30 (523)	14.38±0.27 (8)	<0.0001		qq
		054	14.63±0.30 (523)	ns	0.20		q_
		058	14.63±0.30 (523)	14.63±0.27 (60)	<0.0001		qq
		068	15.29±0.33 (1055)	14.54±0.31 (72)	<0.0001		Qq
		073	15.29±0.33 (1055)	15.29±0.33 (1055)	–		QQ
		074	15.29±0.33 (1055)	14.54±0.31 (72)	<0.0001		Qq
		075	15.29±0.33 (1055)	15.29±0.33 (1055)	–		QQ
		090	14.63±0.30 (523)	14.63±0.30 (523)	–		qq
		094	14.63±0.30 (523)	ns	–		q_
		124	14.63±0.30 (523)	14.63±0.27 (60)	<0.0001		qq
		126	14.63±0.30 (523)	14.63±0.27 (60)	<0.0001		qq
		146	14.63±0.27 (60)	114.38±0.27 (8)	<0.0001		qq
		174	14.63±0.30 (523)	14.89±0.36 (9)	<0.0001		qq
		196	14.63±0.30 (523)	ns	<0.53		q_
		202	15.29±0.33 (1055)	14.63±0.30 (523)	–		Qq
		292	14.63±0.30 (523)	14.5±0.4 (30)	<0.0001		qq
		1190	14.63±0.30 (523)	ns	–		q_
SU	F_0_	5621	13.94±0.29 (52)	13.98±0.27 (49)		−3.87	qq
		5675	13.92±0.19 (51)	13.90±0.20 (39)		−4.96	qq
		6313	14.15±0.22 (47)	14.24±0.24 (38)		−3.05	qq
		6537	14.31±0.27 (42)	14.62±0.28 (50)		2.05	Qq
ET	F_0_	C14	14.14±0.13 (14)	14.70±0.22 (40)	0.001		Qq
	F_1_	C2027	14.28±0.21 (29)	14.67±0.23 (27)	0.003		Qq
		C2042	14.22±0.19 (9)	14.77±0.19 (13)	0.009		Qq
		C2046	14.14±0.13 (14)	14.20±0.18 (10)	0.73		Qq

a WE, White Duroc × Erhualian F_2_ intercross; SU, Sutai pigs; ET, Erhualian × Tongcheng F_2_ intercross.

b Left or right chromosomes are defined arbitrarily for each sire. For left and right chromosomes of F_0_ animals in the two F_2_ intercross populations, *q* chromosomes are judged by the Mendelian inheritance and their significant difference from the average value of deduced *Q* chromosomes in F_1_ sires, or vice versa. Information is not available (ns) for some right chromosomes as no or few F_2_ animals inherit these chromosomes. Some F_0_ animals have identical haplotypes corresponding to *Q* or *q* chromosomes of F1 sires. Average values are shown for these chromosomes.

c The Z-test on 9 F_1_ sires in the White Duroc × Erhualian F_2_ intercross has been reported in our previous study [10].

### An Integrative Analysis Revealed the Most Likely Causal Variants of *VRTN*

**Figure 3:**
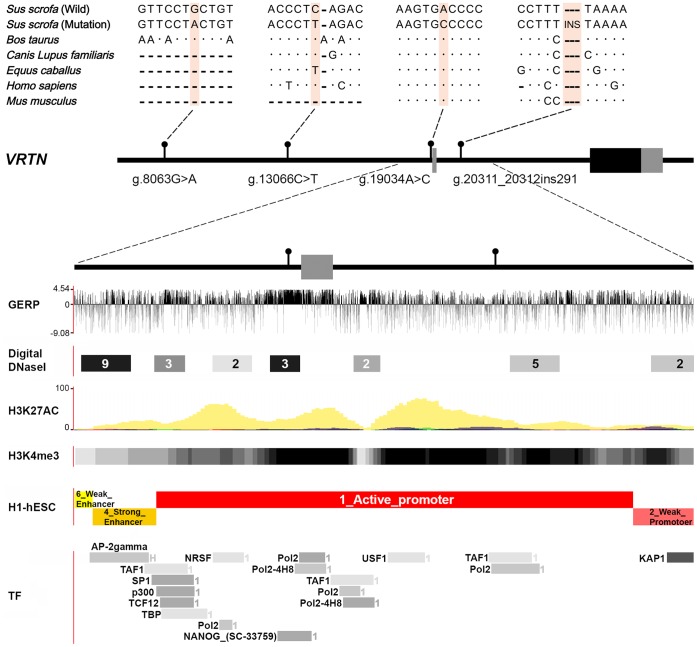
Visualization of evolutionary constraints and functional significance of ***VRTN*** **candidate causal variants.** Four variants showing 100% concordance with the QTL genotypes of parental pigs are shown in the genomic structure of the *VRTN* gene. Exons are indicated by boxes and non-coding regions by thin lines (medium panel). The sequences for the alignment were taken from the following accessions: NC_010449.4 (*Sus scrofa*), AC_000167.1 (*Bos taurus*), NC_006590.3 (*Canis lupus familiaris*), NC_000014.8 (*Homo sapiens*) and NC_000078.6 (*Mus musculus*). The positions of variants are indicated by shaded boxes and the missing sequences are marked by the dash (upper panel). An integrative genomic analysis was performed on a 4-kb region harboring the two most likely causal variants via the UCSC Genome Browser (lower panel). GERP, GERP scores for mammalian alignments; Digital DNaseI, digital DNaseI hypersensitivity clusters; H3K27AC, histone 3 lysine 27 acetylation; H3K4me3, histone 3 lysine 4 trimethylation; H1-hESC, chromatin segmentation by Hidden Markov Model predicted from the ENCODE consortium; TF, transcription binding sites.

To identify the causal variant of *VRTN*, we resequenced a 30-Kb segment covering the *VRTN* gene using the above-mentioned 35 DNA samples. Variants were excluded as causal mutations if their genotypes were discordant with QTL genotypes of the tested animals. Mikawa et al. (2011) [11] have previously reported 9 *VRTN* polymorphisms significantly associated with the number of thoracic vertebrae in European breeds. Of the 9 polymorphic sites, 5 are apparently not causal variants because of the discordance between QTL and mutation genotypes (**Table S3**). The other 4 variants including g.8063G>A, g.13066C>T, g.19034A>C and g.20311_20312ins291 appear to be strong candidate causal variant as their genotypes showed the complete concordance with QTL genotypes across all tested samples (**Table S3**). We did not find any other variants cosegregating with the QTL genotypes in the *VRTN* region. To test evolutionary constraints on the 4 candidate causal variants, we performed the MultiPipMaker alignment of 6 orthologous *VRTN* genes in mammals. Of them, g.8063G>A and g.13066C>T are present in multiple species **(** **Figure 3** **)** and are less likely causal mutations. In contrast, g.19034A>C and g.20311_20312ins291 occur exclusively in domestic pigs, standing out as the promising causal variants. Through the Ensembl Genome Browser, we confirmed the unique occurrence of the two variants in domestic pigs out of 30 eutherian mammals (**Figure S3**). Further, we inferred the functional features of the genomic region encompassing the two variants using the integrative genomic data set on the UCSC Genome Browser. We found that the variants reside in an active promoter. The promoter has typical open chromatin signatures of H3K4me proximal to peaks of H3K27AC that are often found near active regulatory elements. The variants correspond to two transcription factor binding sites with high GERP conservation scores. Moreover, g.19034A>C is located in a DNaseI hypersensitive site that are often associated with transcriptional activity (**Figure 3**). Altogether, these findings form the hypothesis that g.19034A>C and g.20311_20312ins291 are functional variants altering the expression of the porcine *VRTN* gene, presumably leading to the QTL effect on vertebral number.

### Association Analysis in a Large Scale Outbred Samples Support Two *VRTN* Variants as the Causal Mutation(s)

We genotyped g.19034A>C and g.20311_20312ins291 on 435 Sutai pigs and 192 unrelated animals from Duroc, Landrace and Large White. The two variants showed the complete linkage disequilibrium with identical genotypes on all tested animals (data not shown). For subsequent analyses, we focused on g.20311_20312ins291 due to its cost-effective genotyping. To confirm the effect of *VRTN* variants on vertebral number, we further genotyped g.20311_20312ins291 on Sutai pigs (n = 435) and a large scale samples of Western commercial hybrid pigs (n = 1403). Association analysis revealed that this polymorphic site had strong effect on the number of thoracic vertebrae but not the number of lumbar vertebrae (**Table 2**). *Ins*/*ins* (*QQ*) individuals have approximate one additional thoracic segment compared with wide-type homozygotes in both Chinese Sutai and Western hybrid pigs (**Table 2**). This is in agreement with the observed QTL effect, reinforcing the assumption that the *VRTN* variant(s) cause the QTL effect on vertebral number on SSC7.

**Table 2:** Association of *VRTN* most likely causal variants with vertebral number in Sutai and Western commercial hybrid pig populations^a^.

Population	Trait	Genotype (no. of individuals)			Log(1/P*)*	*F-*value
		*ins*/*ins*	i*ns*/−	−/−		
Sutai	thoracic vertebral number	15.07±0.13^a^ (30)	14.35±0.24^b^ (192)	13.88±0.19^c^ (213)	>16	115.80
	lumbar vertebral number	5.27±0.20^a^ (30)	5.45±0.25^b^ (192)	5.49±0.25^b^ (213)	0.07	2.59
DLL^b^	thoracic vertebral number	15.63±0.41^a^ (527)	15.11±0.44^b^ (662)	14.71±0.52^c^ (214)	>16	183.90
	lumbar vertebral number	5.36±0.23^a^ (527)	5.47±0.25^b^ (662)	5.47±0.25^b^ (214)	1.14	3.23

a Phenotypic values are shown in mean ± standard deviation. Values with different superscripts in the same line are significantly different.

b Western three-way hybrid pigs from Duroc × (Landrace × Large White).

### Both Western and Chinese Pigs are Segregating for the *VRTN* Causal Mutation(s)

To investigate the distribution of the *VRTN* causal mutation in a broad panel of breeds, we genotyped g.20311_20312ins291 on 1371 unrelated pigs representing 20 diverse breeds and wild boars (**Table 3**). As expected, wild boars are all homozygous for the wild-type allele as they uniformly have 19 thoracolumbar vertebrae. Most of Chinese indigenous breeds are also fixed at the wild-type allele, which is consistent with the fidelity of the thoracic vertebral number in these breeds. However, the mutation are segregating in several Chinese breeds including Bamaxiang, Erhualian, Hang, Laiwu Black, Tibetan and Tongcheng pigs. Especially, the derived allele for increased vertebral number is present in Hang (29%) and Tongcheng (28%) pigs at rather high frequencies (**Table 3**). This finding, together with the absence of the mutant allele in Chinese wild boars, would suggest that the mutation occurred in China after domestication. The mutation in Western present-day breeds is very common. Interestingly, the highest frequency (71%) of the mutation is found in Landrace that is known for long body length.

**Table 3:** Frequencies of the derived (*Q*) allele for increased vertebral number in Chinese and Western pig breeds.

Breed	Origin	No.	Allele frequency
Chinese breeds			
Bama Xiang	Guangxi	31	0.15
Erhualian	Jiangsu	205	0.06
Hang	Jiangxi	31	0.29
Hetao Big-Ear	Inner Mongolia	30	0.00
Jiangquhai	Jiangsu	32	0.00
Jinhua	Zhejiang	32	0.00
Laiwu Black	Shandong	32	0.14
Lantang	Guangdong	30	0.00
Meishan	Jiangsu	30	0.00
Minzhu	Heilongjiang	30	0.00
Ningxiang	Hunan	32	0.00
Rongchang	Chongqing	32	0.00
Tibetan	Tibet	30	0.08
Tongcheng	Hubei	32	0.28
Wuzhishan pig	Hainan	30	0.00
Yushan Black	Jiangxi	31	0.00
Chinese wild boar	Jiangxi	31	0.00
Western breeds			
Duroc	USA	103	0.54
Landrace	Denmark	175	0.71
Large White	UK, Canada, France	392	0.66

## Discussion

### The QTL for Vertebral Number on SSC7 in Western and Chinese Pigs is Caused by a Common Variant

Western commercial pigs and a number of Chinese indigenous pigs show considerable variation in the thoracic-lumbar vertebral number. To address if common or distinct variants cause numeric variation in vertebral number, we herein performed GWAS mapping of the loci for the phenotypic trait using both Western × Chinese (White Duroc × Erhualian F_2_ cross and Sutai pigs) and Chinese × Chinese (Erhualian × Tongcheng F_2_ intercross) hybrid populations. As a result, we consistently detected genome-wide significant loci for the number of thoracic vertebrae on SSC7 across the three experimental populations, where the most significant SNPs were located in a proximal region (**Figure 1**). This allows us to hypothesize that a common variant causes the major QTL effect in both Western and Chinese pigs. With this assumption, we defined the most likely region of 947-Kb harboring the responsible gene by determining the overlapping segment between the 95% CIs in the 3 tested populations. In the critical region, all *Q*-chromosomes of parental pigs for increased vertebral number share a 100-Kb haplotype containing 15 polymorphic sites. These *Q* chromosomes were originated from three Erhualian founder sows and two White Duroc founder boars in the White Duroc × Erhualian intercross as well as one Tongcheng founder sow in the Erhualian × Tongcheng cross. The shared *Q*-segment across diverse chromosomes of different origins strengths our assumption that the number-increasing allele at the SSC7 QTL in Chinese and Western pigs is derived from a common ancestor. After identifying the most likely causal mutation(s) for the QTL, we performed a comprehensive survey of the genetic variability at the locus in diverse breeds. We found that the mutation is segregating in a number of Chinese breeds (**Table 3**), thus supporting the hypothesis that the QTL is likely of Chinese origin.

According to historical records [23], [24], Chinese indigenous pigs were intercrossed into European pigs approximately 200 years ago, contributing to the formation of Western present-day commercial breeds. This has been confirmed by the recently completed analyses of pig genome that showed a strong signal (37% fraction) of admixture from Chinese breeds into European breeds [25]. It thus raises the possibility that the number-increasing allele was introduced from Chinese pigs into Western pigs at that time. Recombination events in the past two centuries have gradually eroded the introduced Chinese haplotype, leading to the current ‘compound’ (mosaic) haplotype from European and Chinese descent in Western pigs, like White Duroc boars in this study (**Figure 2**). During the past decades, Western modern breeds have experienced intensive selection on carcass length and lean production. It, we believe, dramatically increase the frequency of the causal mutation in these breeds.

It should be noted that we also detected a genome-wide significant locus for vertebral number on SSC1 (**Figure 1**). The locus perfectly corresponds to the *NR6A1* region that has been associated with vertebral number [11]. In contrast to the SSC7 locus, the number-increase allele at the *NR6A1* locus is of European origin. Moreover, the *NR6A1* region shows strong signatures of selection and the beneficial allele has been fixed in European breeds [26].

### Our Findings Support that *VRTN* is the Responsible Gene for the QTL on SSC7

In a previous report, Mikawa et al. (2011) [14] identified a strong candidate gene of the QTL on SSC7 using QTL and linkage disequilibrium mapping studies on European commercial pigs. The previously uncharacterized gene, namely *VRTN*, showed significant association with the number of thoracic vertebrae in Western pigs. We herein used genome-wide 60 K SNPs to conduct GWAS mapping of the QTL on large scale samples from three different populations. By the GWAS and IBD sharing analyses, we define the QTL within a critical region of 100 Kb that harbors only two annotated genes, i.e. *VRTN* and *SYNDIG1L*. *SYNDIG1* is a candidate for Huntington disease in rodent models [22]. It is thus reasonable to deduce that *VRTN* is the responsible gene for the variation in vertebral number at the SSC7 QTL. One paralogue of the *VRTN* gene is located at 103.2 Mb, adjacent to the 100-Kb critical region. The paralogous gene is not likely the underlying gene as it falls outside the IBD region. The orthologs of *VRTN* appear to be a conserved protein in a wide range of organisms from fishes to mammals on the UCSC Genome Browser. VRTN is therefore likely to be an essential regulator for somitogenesis in the development of embryo in a wide range of organisms. Nevertheless, the detailed underlying mechanism of *VRTN* in the development of swine vertebral formula remains largely unknown at present and needs further investigation.

### *VRTN* g.19034A>C and g.20311_20312ins291 are the Most Likely Causal Variants underlying the QTL on SSC7

By resequencing a 41-Kb region around the *VRTN* gene, Mikawa et al. (2011) identified 9 variants that show concordance with the QTL genotypes of family patriarchs and are significantly associated with vertebral number in Western pigs. These variants are in a complete linkage disequilibrium phase. Therefore, the causative polymorphic site could not be defined in the previous study [14]. To resolve the problem, we herein resequenced the *VRTN* gene and applied the concordance test for all polymorphisms including the 9 variants on 35 individuals with deduced QTL genotypes by progeny testing. Notably, four *VRTN* polymorphisms are in complete concordance with the QTL genotypes of the tested animals. All 12 individuals heterozygous for the QTL are also heterozygous for the polymorphisms, and all 23 pigs homozygous for the QTL are also homozygous for the polymorphisms. The finding apparently indicates that these polymorphisms are strong candidate causal variants underlying the QTL.

The number of thoracolumbar vertebrae is always fixed at 19 in mammals [27]. In domestic animals, the changes in thoracolumbar vertebral number are common in pigs and sheep but are very rare in other species [28]. The developmental constraint supports the hypothesis that the underlying mutation for vertebral number in this study is lineage-specific in pigs or sheep among mammals. Of the 4 candidate mutations, g.19034A>C and g.20311_20312ins291 show evolutionary constraints as they exist exclusively in domestic pigs in 30 eutherian mammals. Moreover, the two variants reside in functional elements of an active promoter and likely affect the expression of *VRTN*, as corroborated by diverse transcriptomic and epigenomic data. Therefore, the two variants are more promising causal mutations compared with the other two non-conservative ones. Given the complete linkage disequilibrium between the two polymorphisms, we can not genetically judge if they act collaboratively or one of them is the underlying mutation contributing to the phenotypic variation. Further functional assays are required to address the question.

We genotyped g.20311_20312ins291 on all animals across the two intercross pedigrees and a large sample of pigs from two outbred populations, i.e. Chinese Sutai pigs and Western three-way hybrid pigs. The variant absorbed totally the QTL effect in the intercross populations as all significant SNPs vanished when it was included as a fixed effect in the GWAS statistic model (data not shown). Moreover, the variant affects one additional thoracic vertebra in both two outbred populations, which is in good agreement with the observed QTL effect. The finding again favors the assumption of the variant as the causal mutation. Our robust result based on large scale samples is important for the pig industry as it provides an accurate and efficient diagnostic tool for the genetic improvement of vertebral number in the swine breeding programs.

### Conclusion

By GWAS and IBD mapping studies on three distinct populations, we gave additional supporting evidence for the assumption that *VRTN* is the responsible gene underlying the QTL for the number of thoracic vertebrae on SSC7. We further showed that a common variant determine the QTL effect in both Western and Chinese pigs and the derived allele for increased vertebral number is likely of Chinese origin. Using an integrative analysis of concordance test, evolutionary constraint and deleteriousness prediction, we identified the most likely causal variants in the *VRTN* gene. We confirmed the major effect of the variants on thoracic vertebrae formula in two large scale outbred populations, and revealed the genetic variability at the mutation site in a wide panel of pig breeds. Our findings advance the understanding of the developmental biology of vertebrae formula and establish a reliable breeding tool for thoracic vertebral number in both Chinese and Western pigs. Further studies are warranted to elucidate the underlying molecular mechanism of the *VRTN* causal mutation(s).

## Supporting Information

Figure S1 **Electrophoresis patterns of the** ***VRTN*** **g.20311_20312 ins291 marker.** A direct PCR was performed to diagnose the indel marker as described in Materials and Methods. Lane 1, 3 and 4, −/−; lane 2, *ins*/−; lane 5, *ins*/*ins*. M is 50 bp DNA ladder.
(TIF)

Figure S2 **GWAS for the number of lumbar vertebrae.** GWAS were performed on the White Duroc × Erhualian F_2_ intercross (A), Sutai pigs (B) and Erhualian × Tongcheng F_2_ intercross (C). Negative log10 *P*-values of all SNPs are plotted against position on each pig chromosome in the y-axis. Chromosomes are shown in different colors for clarity in the x-axis. Log (1/*P*) values of more than 5 are genome-wide significant.
(TIF)

Figure S3 **Conservation of the chromosomal region encompassing the** ***VRTN*** **most likely causal variant (g.19034A>C) in 30 eutherian mammals.** For clarity, the sole occurrence of g.20311_20312 ins291 in these mammals is not shown in this figure.
(TIF)

Table S1 **PCR primers for the characterization of polymorphisms in the IBD region and the resequencing of the porcine** ***VRTN*** **gene.**
(DOC)

Table S2 **The effect of the most significant SNPs on the number of thoracic vertebrae in three experimental populations.**
(DOC)

Table S3 **Concordance of** ***VRTN*** **candidate causal variants and QTL genotypes of parental pigs in three experimental populations.**
(DOC)
