# Supplementary figures and images for: A Further Look at Porcine Chromosome 7 Reveals VRTN Variants Associated with Vertebral Number in Chinese and Western Pigs
Source: PLoS One. 2013 Apr 24;8(4):e62534. doi: 10.1371/journal.pone.0062534 (PMC3634791; doi:10.1371/journal.pone.0062534)

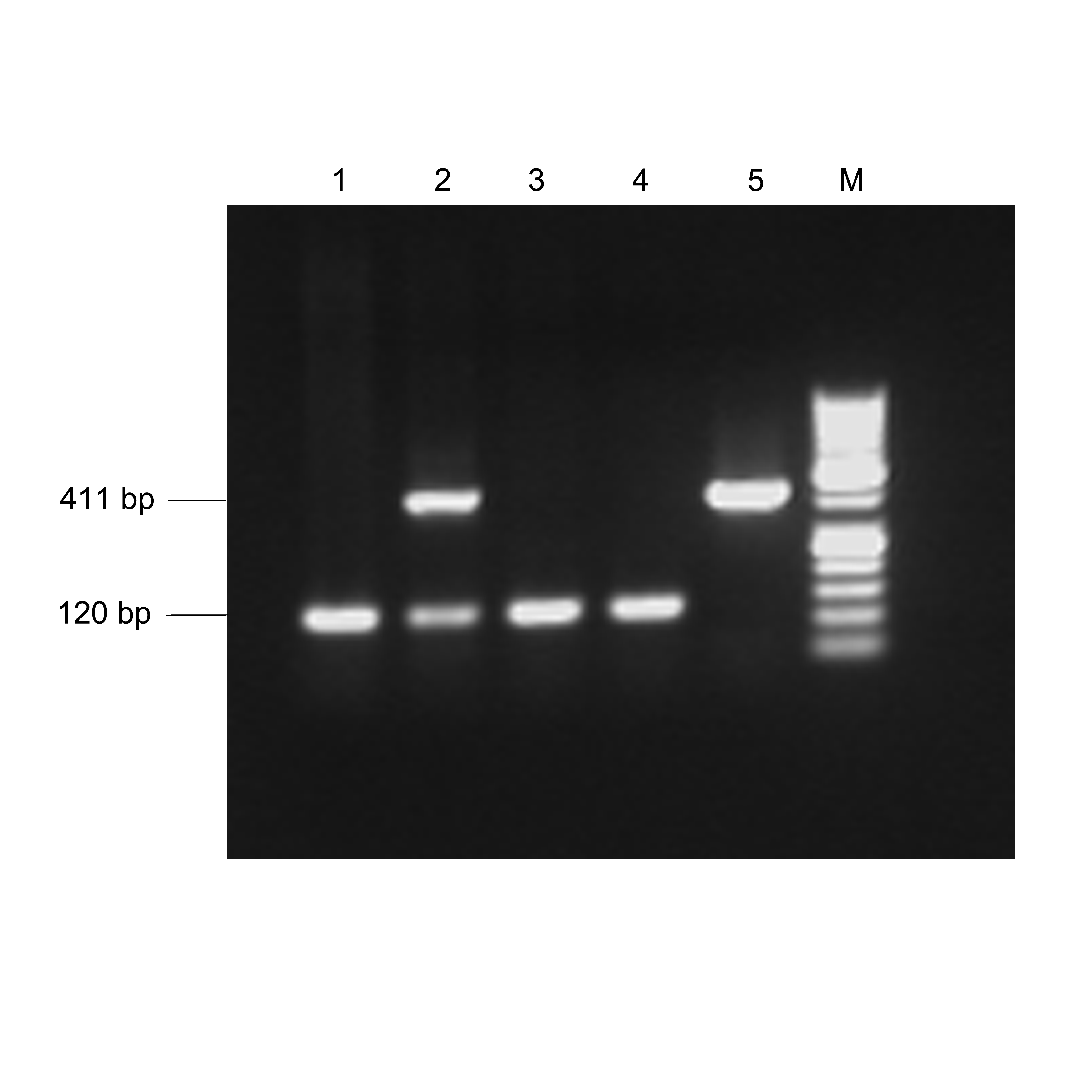

Supplement: Figure S1 — Electrophoresis patterns of the VRTN g.20311_20312 ins291 marker. A direct PCR was performed to diagnose the indel marker as described in Materials and Methods. Lane 1, 3 and 4, −/−; lane 2, ins/−; lane 5, ins/ins. M is 50 bp DNA ladder. (TIF) [file pone.0062534.s001.tif]

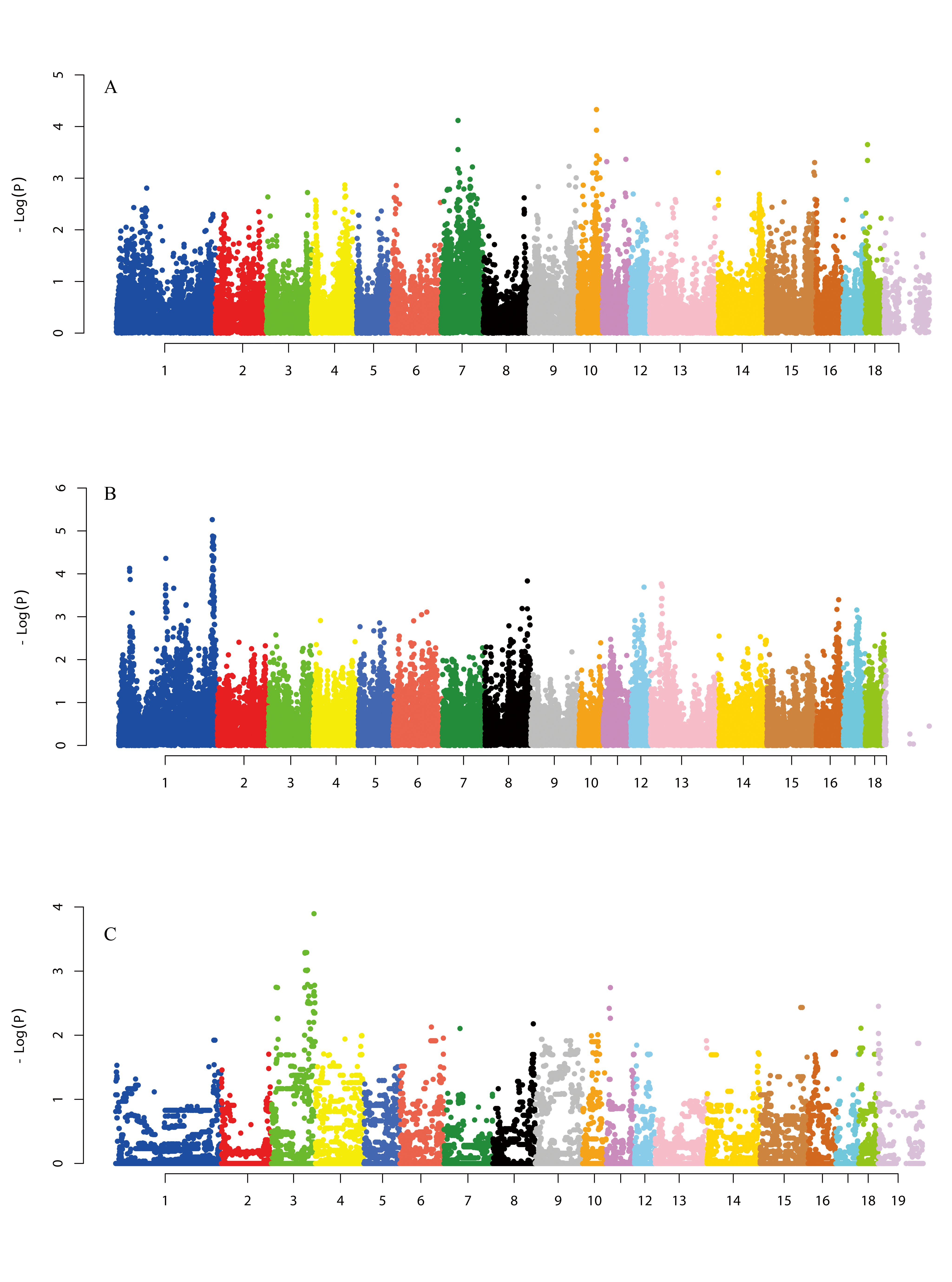

Supplement: Figure S2 — GWAS for the number of lumbar vertebrae. GWAS were performed on the White Duroc × Erhualian F2 intercross (A), Sutai pigs (B) and Erhualian × Tongcheng F2 intercross (C). Negative log10 P-values of all SNPs are plotted against position on each pig chromosome in the y-axis. Chromosomes are shown in different colors for clarity in the x-axis. Log (1/P) values of more than 5 are genome-wide significant. (TIF) [file pone.0062534.s002.tif]

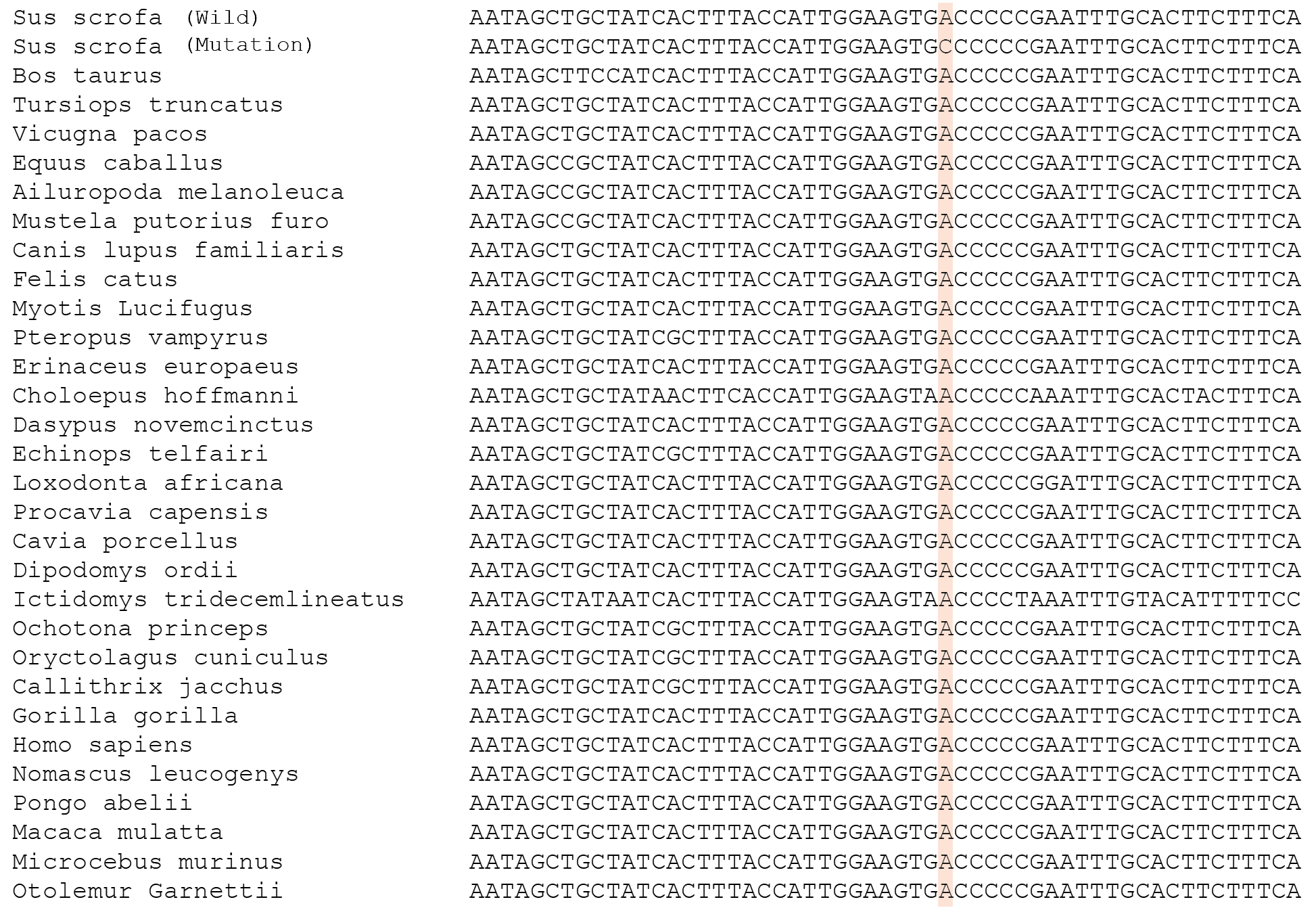

Supplement: Figure S3 — Conservation of the chromosomal region encompassing the VRTN most likely causal variant (g.19034A>C) in 30 eutherian mammals. For clarity, the sole occurrence of g.20311_20312 ins291 in these mammals is not shown in this figure. (TIF) [file pone.0062534.s003.tif]
