# Supplementary material for: A Further Look at Porcine Chromosome 7 Reveals VRTN Variants Associated with Vertebral Number in Chinese and Western Pigs
Source: PLoS One. 2013 Apr 24;8(4):e62534. doi: 10.1371/journal.pone.0062534 (PMC3634791; doi:10.1371/journal.pone.0062534)
Supplement: Table S1 — PCR primers for the characterization of polymorphisms in the IBD region and the resequencing of the porcine VRTN gene. (DOC) [file pone.0062534.s004.doc]

**Table S1. PCR primers for the characterization of polymorphisms in the IBD region and the resequencing of the porcine *VRTN* gene ^a^.**

| Polymorphism | Primer | Sequence (5’-3’) | Amplicon (bp) |
| --- | --- | --- | --- |
| g.8063G>A | F1 | TGTGCATTGTAAAACATAAAAACTGA | 173 |
|  | R2 | GCAGCAGCTTCTTCAGCAAT |  |
| g.11501A>T | F2 | ATTTGGCCTTGGTCTTGCCT | 183 |
|  | R2 | GGGAAGAAAGCGTGTGGATA |  |
| g.13066C>T | F3 | GGGGAGGAAACTGAGTGTTGG | 645 |
|  | R3 | TGTCCGATTTCACTGTCTTCC |  |
| g.16647A>T | F4 | CGGCTTCTGGAAAGAGAAAA | 187 |
|  | R4 | AACTGTGGTTTCAGGCATCC |  |
| g.19034 A>C | F5 | GGGTAGAGTGGACGAGACAATAGC | 79 |
|  | R5 | CCGAGATGAAAGAAGTGCAAATT |  |
|  | Probe 1 | CATTGGAAGTGCCC |  |
|  | Probe 2 | TACCATTGGAAGTGACC |  |
| g.20311_20312 ins291 | F6 | GGCAGGGAAGGTGTTTGTTA | 411, 120 |
|  | R6 | GACTGGCCTCTGTCCCTTG |  |
| g.24802_24805insaa | F7 | TTTAACTTGCAGGCCCTCTG | 157 |
|  | R7 | CTAGCATGGGACCCTCCATA |  |
| g.36291del C | F8 | TTGTCTGGGGAGTAGGGTTG | 177 |
|  | R8 | AACCTGGTTCGATCTTAATTTCA |  |
| g.41709A>C | F9 | AGAGGCCTTGGAGGTAAAGG | 167 |
|  | R9 | CATAGGGTCTGCCAGGGTGT |  |
| 103370106T>A | F10 | GCACAAAGCAAGTCCTCTAAG | 635 |
|  | R10 | ACCTCCTCAGGGTTACTAATCA |  |
| 103381467A>G,103381542T>C,  103381576A>G,103381663T>C,  103381725T>C,103381764T>C | F11 | AGATGTCTCTGAGGTAGGAAGC | 687 |
|  | R11 | CAAGAACTCACACACCGTATTT |  |
|  |  |  |  |
| 103395139A>G,103395541T>G | F12 | CCAGATTCAACTTGGAAAGTCT | 650 |
|  | R12 | GGAGAGGTGTATGGACAGGTAT |  |
| 103407127G>A,103407193A>C,  103407222G>T,103407263A>G | F13 | TCAGACAGACTCACCATAGCTC | 619 |
|  | R13 | CTCTACCTGGTCCTCCTCTTC |  |
| 103434915T>C,103434951G>A | F14 | GGTGCTCATTAAACACTTCTGA | 605 |
|  | R14 | TTTGAAACTGCTGTATCTCTGG |  |
| 103451019T>G,103451132T>G,  103451235C>T | F15 | CTATTTTACATACAGGGGAGGA | 700 |
|  | R15 | TTAGCACAGGTCTCAGAATATG |  |
| 103464554A>G | F16 | ACATGGGTATCTTGTCCTGA | 627 |
|  | R16 | TGGGGTAGATGGAGTAGATG |  |
| 103475125A>T,103475166A>G,  103475319A>C | F17 | CGGACTGTATTATACCCTGAAT | 630 |
|  | R17 | TTCCTAGAGTTCTCCTAAGTGG |  |
| 103489954G>A,103489985T>G,  103490055G>T,103490134A>G | F18 | TGTTATACAAGAGAAGCCTTGG | 634 |
|  | R18 | AAAGCCTGTGTAGCTTCAATAC |  |
| 103506424T>C | F19 | CATACCTTCTGAGTTTCCTCAT | 655 |
|  | R19 | GACCACATGTTCTTCTTTGTG |  |
| 103537491A>G | F20 | CAGAACAAGTGAGGTAGTTCAA | 658 |
|  | R20 | TTGAGGCTCAGATAGGTTAAGT |  |
| 103552163G>A,103552439C>T,  103552550G>T,103552572A>G | F21 | CTCTGCAAAGTTTAGGATCTTC | 635 |
|  | R21 | ATGACACAGATACATTCACACC |  |
| 103572282C>T,103572407G>T | F22 | AGCTTAGCAACATGAGTCTTCT | 664 |
|  | R22 | CCCACTAATTTCACTAGATTCC |  |
| ssc7-vet-01-FP | F23 | ATAAAGAGTAGCAGAAGTGGGGCTGA | 1833 |
| ssc7-vet-01-RP | R23 | AGCACTGGCTCAGAACCTAATAAACG |  |
| ssc7-vet-02-FP | F24 | TGAACCTGAGTCCTCATAGACACGAG | 2039 |
| ssc7-vet-02-RP | R24 | CATCTTCTTGATCCACTCCTCTTGGT |  |
| ssc7-vet-03-FP | F25 | TGACAACTCCCAGACTCACTTCACTC | 2144 |
| ssc7-vet-03-RP | R25 | TCGGATTCGTTTCCACTACACTACAA |  |
| ssc7-vet-04-FP | F26 | TAAAACACACCCAAACTGAGAGAATGG | 2000 |
| ssc7-vet-04-RP | R26 | CTGCGTGCACTTGAACAAGTTTTCT |  |
| ssc7-vet-05-FP | F27 | AATGAGAAGGGAAGACAGTGAAATCG | 1918 |
| ssc7-vet-05-RP | R27 | GCTAAAGCCTTTTCCTTCTTTTGCTC |  |
| ssc7-vet-06-FP | F28 | GGGCAAAGGATATTCATAGGAAAGGA | 2134 |
| ssc7-vet-06-RP | R28 | CATATCTGCAGTGTGGCTTTCTTTGT |  |
| ssc7-vet-07-FP | F29 | GCTTCTGGAAAGAGAAAACTGTGGAC | 2003 |
| ssc7-vet-07-RP | R29 | ACAAAGATGCTTTTGTCAGGGGTCAG |  |
| ssc7-vet-08-FP | F30 | GGTGATGAGTGTAGGTTCATGACTGG | 1962 |
| ssc7-vet-08-RP | R30 | TCAGGAAGACTGGCTAAGACTGTGGT |  |
| ssc7-vet-09-FP | F31 | CCAGACATGTGGTCCACTAGAGCTAC | 2226, 1935 |
| ssc7-vet-09-RP | R31 | TCGCCACCATTTACCTACTGCTTACT |  |
| ssc7-vet-10-FP | F32 | CAAGCCTCTGATTTCTCAAGGGTAAA | 2028 |
| ssc7-vet-10-RP | R32 | AGGAACTACCAGCTGTCAACTTTTGG |  |
| ssc7-vet-11-FP | F33 | AGATCCCACCTGTCTCTCTGTTCAGT | 2112 |
| ssc7-vet-11-RP | R33 | CGTTTGCATCTACTAACCCCAAACTC |  |
| ssc7-vet-12-FP | F34 | TCCAGGCAGACCAATTTTAGCTATGT | 1991 |
| ssc7-vet-12-RP | R34 | CGTGCTTCCAGGCATAATAGGTAGAG |  |
| ssc7-vet-13-FP | F35 | CCTCAGCTACTCCTACCTCTGTGAGC | 2060 |
| ssc7-vet-13-RP | R35 | AGGCTCTGAGGAGGGACTTAACAGAA |  |
| ssc7-vet-14-FP | F36 | AGAAGTGGCGATGTTACCAGAAGAAC | 2377 |
| ssc7-vet-14-RP | R36 | CACACTAACAAGCCTTCCCAGAGACT |  |
| ssc7-vet-15-FP | F37 | TTCTAGGCTTATTTCCTCCCTCAACC | 2271 |
| ssc7-vet-15-RP | R37 | AAATTCTAGGATGAAGCCCAGCTTTC |  |
| ssc7-vet-16-FP | F38 | CAACCCACCTGCTCTTCTCTCAGTAT | 2460 |
| ssc7-vet-16-RP | R38 | AGCTTTGTATCCTTCCTTTCCCTCAC |  |
| ssc7-vet-17-FP | F39 | CGGTCCTAGAAAAGGCAAAAAGAGAT | 2238 |
| ssc7-vet-17-RP | R39 | AGCGATCAAACTCACTGCTCAGTATC |  |
| ssc7-vet-18-FP | F40 | TTCTTGGAGAGTTAGCAGTGATGTGC | 2223 |
| ssc7-vet-18-RP | R40 | GACAGGAACTCCCTTCTGTGAACATT |  |
| ssc7-vet-19-FP | F41 | TCTACCCTATGACCCACCATTTTCAG | 2228 |
| ssc7-vet-19-RP | R41 | GGCCAGACCAGTAATCTTCTTTGACA |  |
| ssc7-vet-20-FP | F42 | CTGGGTCTTGCTTAAAATGTACCACA | 2377 |
| ssc7-vet-20-RP | R42 | TAGCAAGAACTCCAAGTCACCCTTTC |  |

^a^ The polymorphisms corresponding to primers F1/R1 to F9/R9 are defined according to GenBank accession no. AB554652.1. The remaining polymorphisms are nominated on the basis of their locations on the pig genome assembly (Sscrofa10.2).
