# Supplementary material for: A Further Look at Porcine Chromosome 7 Reveals VRTN Variants Associated with Vertebral Number in Chinese and Western Pigs
Source: PLoS One. 2013 Apr 24;8(4):e62534. doi: 10.1371/journal.pone.0062534 (PMC3634791; doi:10.1371/journal.pone.0062534)
Supplement: Table S2 — The effect of the most significant SNPs on the number of thoracic vertebrae in three experimental populations. (DOC) [file pone.0062534.s005.doc]

**Table S2 The effect of the most significant SNPs on the number of thoracic vertebrae in three experimental populations ^a^.**

| Population | Genotype (no. of individuals) | | | Log(1/P*)* | *F-*value |
| --- | --- | --- | --- | --- | --- |
|  | 11 | 12 | 22 |  |  |
| White Duroc × Erhualian F_2_ intercross | 15.48 ± 0.03 ^a^ (341) | 14.91 ± 0.04 ^b^ (446) | 14.17 ± 0.05 ^c^ (129) | 34.11 | 357.70 |
| Sutai pigs | 15.04 ± 0.09 ^a^ (26) | 14.40 ± 0.10 ^b^ (171) | 13.87 ± 0.09 ^c^ (211) | 21.25 | 115.40 |
| Tongcheng × Erhualian F_2_ intercross | 14.83 ± 0.09 ^a^ (12) | 14.66 ± 0.13 ^b^ (12) | 14.00 ± 0.11 ^c^ (20) | 4.90 | 29.82 |

^a^ Phenotypic values are shown in mean ± standard deviation. Values with different superscripts in the same line are significantly different.
