# Supplementary material for: A Further Look at Porcine Chromosome 7 Reveals VRTN Variants Associated with Vertebral Number in Chinese and Western Pigs
Source: PLoS One. 2013 Apr 24;8(4):e62534. doi: 10.1371/journal.pone.0062534 (PMC3634791; doi:10.1371/journal.pone.0062534)
Supplement: Table S3 — Concordance of VRTN candidate causal variants and QTL genotypes of parental pigs in three experimental populations. (DOC) [file pone.0062534.s006.doc]

**Table S3.** Concordance of *VRTN* candidate causal variants and QTL genotypes of parental pigs in three experimental populations.

| Population | Individual | Generation | QTL genotype | Mutation genotype | | | | | | | | |
| --- | --- | --- | --- | --- | --- | --- | --- | --- | --- | --- | --- | --- |
|  |  |  |  | g.8063  G>A | g.11051  A>T | g.13066  C>T | g.16647  A>T | g.19034  A>C | g.20311_20312  ins291 | g.24802_24805  insaa | g.36291  delC | g.41709  A>C |
| WE | 73 | F0 | QQ | AA | TT | TT | TT | CC | ins/ins | AAA/AAA | DEL/DEL | CC |
|  | 75 | F0 | QQ | AA | TT | TT | TT | CC | ins/ins | AAA/AA | DEL/DEL | CC |
|  | 38 | F0 | qq | GG | AA | CC | *tt* | AA | -/- | AAAAA/AAAAA | CC | AA |
|  | 52 | F0 | qq | GG | *at* | CC | AA | AA | -/- | AAAAA/AAAAA | CC | AA |
|  | 54 | F0 | q_ | GG | AT | CC | AA | AA | -/- | AAAAA/AAAAA | CC | AA |
|  | 58 | F0 | qq | GG | *at* | CC | AA | AA | -/- | AAAAA/AAAAA | CC | AA |
|  | 68 | F0 | Qq | GA | *tt* | CT | *tt* | AC | ins/- | *aaa/aaa* | DEL/C | *cc* |
|  | 74 | F0 | Qq | AG | *tt* | CT | *tt* | AC | ins/- | *aaa/aaa* | DEL/C | *cc* |
|  | 90 | F0 | qq | GG | *at* | CC | AA | AA | -/- | AAAAA/AAAAA | CC | AA |
|  | 94 | F0 | q_ | GG | AA | CC | AA | AA | -/- | AAAAA/AAAAA | CC | AA |
|  | 124 | F0 | qq | GG | AA | CC | AA | AA | -/- | AAAAA/AAAAA | CC | AA |
|  | 126 | F0 | qq | GG | AA | CC | AA | AA | -/- | AAAAA/AAAAA | CC | AA |
|  | 146 | F0 | qq | GG | AA | CC | AA | AA | -/- | AAAAA/AAAAA | CC | AA |
|  | 174 | F0 | qq | GG | AA | CC | AA | AA | -/- | AAAAA/AAAAA | CC | AA |
|  | 196 | F0 | q_ | GG | AT | CC | AA | AA | -/- | *aaa/aaaaa* | CC | AA |
|  | 202 | F0 | Qq | GA | AT | CT | *aa* | AC | ins/- | AAA/AAAAA | DEL/C | *cc* |
|  | 292 | F0 | qq | GG | *at* | CC | / | AA | -/- | AAAAA/AAAAA | CC | AA |
|  | 1190 | F0 | q_ | GG | AA | CC | AA | AA | -/- | AAAAA/AAAAA | CC | AA |
|  | 3 | F1 | Qq | GA | AT | CT | *tt* | AC | ins/- | AAA/AAAAA | DEL/C | *cc* |
|  | 17 | F1 | Qq | GA | AT | CT | TA | AC | ins/- | AAA/AAAAA | *del/del* | *cc* |
|  | 23 | F1 | QQ | AA | TT | TT | TT | CC | ins/ins | AAA/AAA | DEL/DEL | CC |
|  | 29 | F1 | QQ | AA | TT | TT | TT | CC | ins/ins | AAA/AAA | DEL/DEL | CC |
|  | 35 | F1 | Qq | GA | *tt* | CT | *tt* | AC | ins/- | *aaa/aaa* | DEL/C | *cc* |
|  | 41 | F1 | Qq | GA | AT | CT | *tt* | AC | ins/- | AAA/AAAAA | DEL/C | *cc* |
|  | 47 | F1 | QQ | AA | TT | TT | TT | CC | ins/ins | AAA/AAA | DEL/DEL | CC |
|  | 49 | F1 | Qq | GA | *tt* | CT | *aa* | AC | ins/- | *aaa/aaa* | DEL/C | *cc* |
|  | 75 | F1 | Qq | GA | *tt* | CT | *aa* | AC | ins/- | *aaa/aaa* | DEL/C | *cc* |
| ET | C14 | F0 | Qq | GA | AT | CT | *tt* | AC | ins/- | AAA/AAAAA | DEL/C | *cc* |
|  | C2027 | F1 | Qq | GA | AT | CT | TA | AC | ins/- | AAA/AAAAA | DEL/C | *cc* |
|  | C2042 | F1 | Qq | GA | AT | CT | *tt* | AC | ins/- | AAA/AAAAA | DEL/C | *cc* |
|  | C2046 | F1 | qq | GG | AA | CC | AA | AA | -/- | AAAAA/AAAAA | CC | AA |
| SU | 5621 | F0 | qq | GG | AA | CC | AA | AA | -/- | AAAAA/AAAAA | CC | AA |
|  | 5675 | F0 | qq | GG | AA | CC | AA | AA | -/- | AAAAA/AAAAA | CC | AA |
|  | 6313 | F0 | qq | GG | AA | CC | AA | AA | -/- | AAAAA/AAAAA | CC | AA |
|  | 6537 | F0 | Qq | GA | AT | CT | TA | AC | ins/- | AAA/AAAAA | DEL/C | CA |

The polymorphisms are defined according to GenBank accession number AB554652.1. SNP g.8063 G>A corresponds to NV083 described by Mikawa et al. (2011). The correspondence of the other 8 variants are g.11051A>T (NV149), g.13066C>T (NV090), g.16647A>T (NV024), g.19034A>C (NV027(2)), g.20311_20312ins291 (NV123), g.24802_24805insaa (NV107), g.36291delC (NV067), g.41709A>C (NV071). Mutation genotypes disconcordant with QTL genotypes are indicated by small italic letters. WE, White Duroc × Erhualian F_2_ cross; ET, Erhualian × Tongcheng F_2_ cross. SU, Sutai pigs.
